# Cost-Effective Approaches Based on Machine Learning to Predict Dynamic Modulus of Warm Mix Asphalt with High Reclaimed Asphalt Pavement

**DOI:** 10.3390/ma13153272

**Published:** 2020-07-23

**Authors:** Dong Van Dao, Ngoc-Lan Nguyen, Hai-Bang Ly, Binh Thai Pham, Tien-Thinh Le

**Affiliations:** 1 University of Transport Technology, Hanoi 100000, Vietnam; banglh@utt.edu.vn (H.-B.L.); binhpt@utt.edu.vn (B.T.P.); 2 University of Transport and Communications, Hanoi 100000, Vietnam; nguyenngoclan@utc.edu.vn; 3 Institute of Research and Development, Duy Tan University, Da Nang 550000, Vietnam; letienthinh@duytan.edu.vn

**Keywords:** warm mix asphalt, reclaimed asphalt pavement, dynamic modulus, machine learning

## Abstract

Warm mix asphalt (WMA) technology, taking advantage of reclaimed asphalt pavements, has gained increasing attention from the scientific community. The determination of technical specifications of such a type of asphalt concrete is crucial for pavement design, in which the asphalt concrete dynamic modulus (E*) of elasticity is amongst the most critical parameters. However, the latter could only be determined by complicated, costly, and time-consuming experiments. This paper presents an alternative cost-effective approach to determine the dynamic elastic modulus (E*) of WMA based on various machine learning-based algorithms, namely the artificial neural network (ANN), support vector machine (SVM), Gaussian process regression (GPR), and ensemble boosted trees (Boosted). For this, a total of 300 samples were fabricated by warm mix asphalt technology. The mixtures were prepared with 0%, 20%, 30%, 40%, and 50% content of reclaimed asphalt pavement (RAP) and modified bitumen binder using Sasobit and Zycotherm additives. The dynamic elastic modulus tests were conducted by varying the temperature from 10 °C to 50 °C at different frequencies from 0.1 Hz to 25 Hz. Various common quantitative indications, such as root mean square error (RMSE), mean absolute error (MAE), and correlation coefficient (R) were used to validate and compare the prediction capability of different models. The results showed that machine learning models could accurately predict the dynamic elastic modulus of WMA using up to 50% RAP and fabricated by warm mix asphalt technology. Out of these models, the Boosted algorithm (R = 0.9956) was found as the best predictor compared with those obtained by ANN-LMN (R = 0.9954), SVM (R = 0.9654), and GPR (R= 0.9865). Thus, it could be concluded that Boosted is a promising cost-effective tool for the prediction of the dynamic elastic modulus (E*) of WMA. This study might help in reducing the cost of laboratory experiments for the determination of the dynamic modulus (E*).

## 1. Introduction

Hot Mix Asphalt (HMA) is the most widely used pavement material [1,2,3]. First introduced in the early 1900s, it could be stated that HMA technology has been fully understood to date [1,2,3]. However, the use of HMA has posed severe problems for the environment as HMA is typically produced at temperatures from 140 °C to 160 °C [3]. Therefore, Warm Mix Asphalt (WMA) technology has been proposed and developed in many countries over the past few years to improve the performance of the HMA [3]. The ultimate goal of using WMA is to reduce emissions and better control the quality of asphalt mixtures with a lower average mix temperature from 20 °C to 40 °C compared to HMA [4]. In addition, the use of WMA technology exhibits many advantages including (i) lower energy consumption compared to HMA technology, thus allowing a reduction of 25–35% of energy, (ii) reducing greenhouse gas emissions by 25–40%, and (iii) reducing human exposure to smoke in batching plants and construction sites by 30–50% compared to HMA [5,6,7,8].

In the design of advanced pavement using WMA, the mechanistic-empirical method (ME) has been widely used in the analysis and forecasting of the long-term features of asphalt pavement [9]. Out of these, the dynamic modulus (E*) of the asphalt mixture is one of the most crucial mechanical parameters used in the analysis of WMA’s performance [10]. Even though the ME method can allow forecasting of the E* over time with input parameters related to virgin materials, loads, and weather conditions, its accuracy sometimes is affected by many uncertainty factors related to the experimental conditions (accuracy of types of equipment and experience of testers) [11]. In addition, some empirical equations have also been developed to predict and determine E* in correlation with several influential parameters, such as proposed by Witczak [10] and Christensen et al. [12]. Although these equations provided alternative ways to predict E*, only several fixed parameters were used in the construction of these equations. Thus, it is required to find a better flexible and alternative way for a more accurate prediction of the E*.

In recent years, more advanced and objective machine learning approaches have been developed and applied to predict the mechanical properties of the materials accurately, including asphalt mixtures. As an example, Nivedya et al. [13] developed and used a popular machine learning technique, namely Artificial Neural Networks (ANN), for the prediction of field permeability of HMA pavement layers and they stated that ANN is a promising tool for accurately predicting the field permeability of HMA. In another study, the k-nearest neighbor model—an effective machine learning algorithm—was applied to predict the moisture susceptibility of HMA [14], which suggested that this machine learning model should be used for accurate prediction of the vital properties of the HMA. Androjić et al. [15] studied and compared two popular machine learning models namely ANN and multiple linear regression (MLR) for prediction of different HMA properties (air void and soluble binder content), and concluded that both applied machine learning models are suitable for prediction of the HMA properties. However, ANN is better than MLR at predicting the air void content, whereas MLR is better than ANN in predicting soluble binder content. In general, these machine learning models are useful for the prediction of the properties of the asphalt materials. However, their applications are still limited in WMA materials. 

In this contribution, the main objective was to develop a cost-effective and alternative approach based on machine learning algorithms (ANN, support vector machine—SVM, Gaussian process regression—GPR, and ensemble boosted trees—Boosted) to predict the dynamic modulus (E*) of WMA. For this, the database, including 300 experimental results, was constructed using the laboratory tests supported by the national research project in Vietnam. In the WMA mixtures, reclaimed asphalt pavement (RAP) was used as it is considered an excellent solution to reduce the need for virgin materials. Thus, it could reduce the aging of asphalt binders and reduce construction temperatures due to the reduction of energy consumption as well as greenhouse gas emissions. To validate and compare the models, various quantitative–statistical indices, namely root mean square error (RMSE), mean absolute error (MAE), and correlation coefficient (R) were used. Matlab codes and packages were used for the development and validation of the models. 

## 2. Experimental

### 2.1. Materials

The andesite aggregates used in this research were collected from the Tan Cang plant, Bien Hoa, Dong Nai, Vietnam. Table 1 shows the test results of the aggregates with the maximum aggregate sizes corresponding to 12.5 mm, 9.5 mm, 4.75 mm, and the mineral filler. It is worth noticing that all the aggregate properties met the requirements specified according to AASHTO M323. Two types of additives, namely Sasobit and Zycotherm, were employed to modify the 60/70 grade bitumen using the wet process of mixing. Sasobit and Zycotherm are the two commonly used additives in WMA technology, aiming for the improvement of the bitumen coverage percentage on aggregates, as well as workability for WMA mixtures using RAP. Derived from the gasification process of coal, Sasobit additive has organic origins with long-chain aliphatic hydrocarbons. The working mechanism of Sasobit consists of decreasing the bitumen viscosity, thus increasing the compacting ability of the WMA mixtures. The long-chain of Sasobit is composed of 40 to 115 atoms of carbon. As stated by the producer, the melting point of Sasobit is 100 °C, and complete dissolution in bitumen is reported at a temperature above 115 °C. Moreover, Sasobit additive helps to improve the durability of the WMA mixtures. On the other hand, Zycotherm additive is a commercial product, in the liquid form odorless, and the main composition is nano organosilane. The density of Zycotherm additive is 0.97 (g/cm^3^), whereas the viscosity is in the range of 1 to 5 (Pa.s). The use of Zycotherm additive contributes to the decrease of the surface tension, increasing the bitumen coverage on aggregates at a given mixing temperature, as well as during the construction stage. In addition, Zycotherm additive helps increase the chemical bonding between the bituminous binders and the surface of aggregates. Table 2 shows the testing results of the two types of modified bitumen. Reclaimed Asphalt Pavement (RAP) was used with a nominal maximum aggregate size of 12.5 mm, which has a similar rock origin as the aggregates. The extraction and recovery process of bitumen in RAP followed the AASHTO T319 [16]. The test results of the recovered bitumen recovery process are shown in Table 3.

### 2.2. Sample Design and Preparation

**Figure 1:**
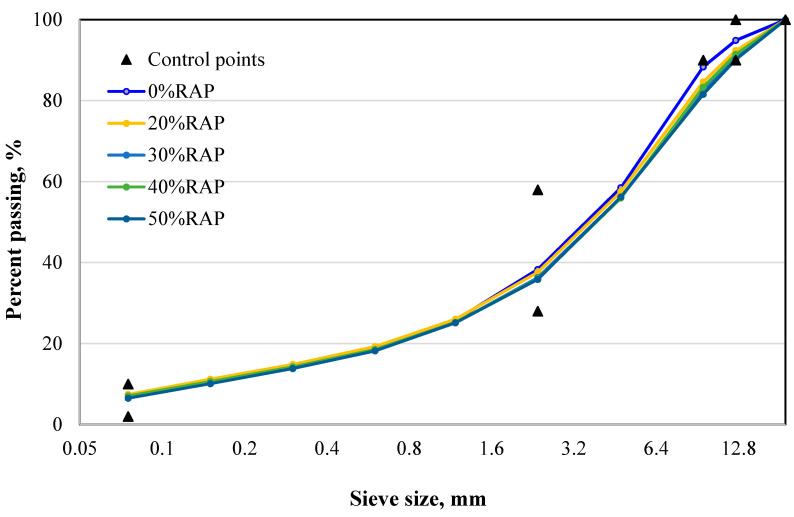
Gradation plot for different % reclaimed asphalt pavement (RAP) mixes.

The mixtures were designed according to the Marshall method [17,18], whereas the aggregate gradation followed the AASHTO M323 [19]. The Sasobit and Zycotherm contents were chosen as 1.5% and 0.15% of the overall weight, as recommended by the producers [4]. The RAP contents were selected as 0%, 20%, 30%, 40%, and 50% of the overall weight. It is worth noticing that the mixture with 0% RAP corresponded to the control mix. Figure 1 shows the aggregate gradation with different RAP contents, according to the AASHTO M323 requirements.

The mixtures were designed according to the Marshall method based on the volumetric properties, the Marshall stability, and the flow criteria. The mixing temperature was 140–145 °C, whereas the compaction temperature was selected as 130–135 °C. The composition and properties of mixtures are shown in Table 4.

### 2.3. Determination of Dynamic Modulus (E*)

**Figure 2:**
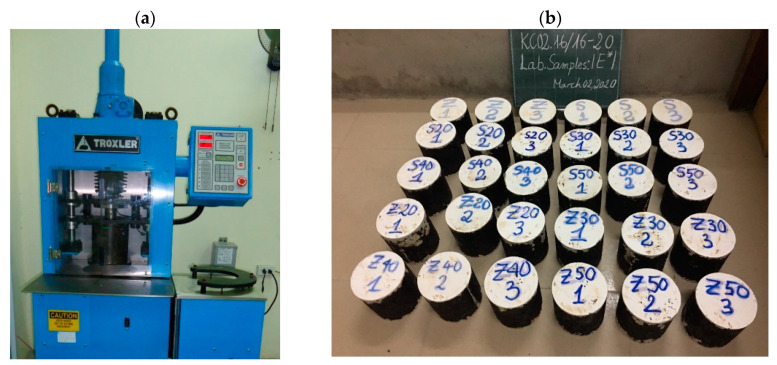
The sample preparation: (**a**) Compaction by Gyratory Compactor machine, (**b**) The testing samples.

The determination of the dynamic modulus (E*) was conducted following the AASHTO TP 62 [20]. The short-term aging of the asphalt concrete, aiming at simulating the oxidation and asphalt absorption into the aggregates, was performed with the guidance of AASHTO R30 [21]. The samples were then compacted using a gyratory compactor in order to achieve the air void value of (V_a_) of 7 ± 0.5%. After compacting, the flattened specimens had a height of 100 mm and a diameter of 100 mm at both ends (Figure 2).

**Figure 3:**
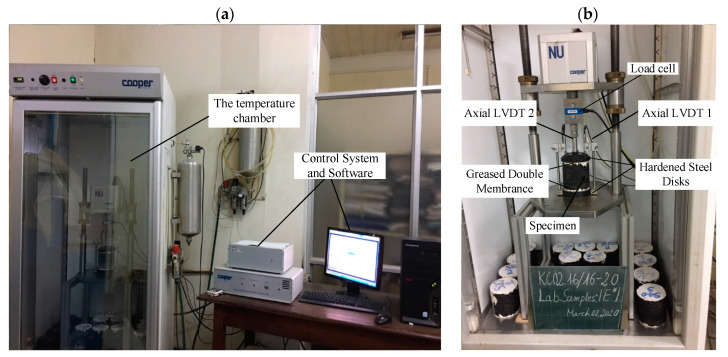
Dynamic Modulus testing: (**a**) The temperature cabinet and control system, (**b**) General Schematic of Dynamic Modulus Test.

The determination of dynamic modulus (E*) was performed in varying the temperature from low to high, combined with a broad range of frequency applied by the load. Precisely, the test temperatures were 10 °C, 20 °C, 30 °C, 40 °C, and 50 °C. At each temperature, the tests were conducted from the highest to lowest frequency, in the order of 25 Hz, 10 Hz, 5 Hz, 1 Hz, 0.5 Hz, and 0.1 Hz. All samples were placed in a thermostatic chamber to maintain a stable temperature before testing, with an error of ±0.1 °C. The curing time for samples was varied from 4 h to 6 h in the thermostatic chamber. Figure 3 shows the dynamic modulus (E*) test device and the sample test.

Overall, a total of 300 experimental results were obtained by taking the average value of 6 samples for each of the above results. The total number of samples fabricated was 1800 samples. The tests contained mixtures with two types of additives (i.e., Zycotherm and Sasobit), five values of %RAP (i.e., 0%, 20%, 30%, 40%, and 50%), six values of testing frequencies (i.e., 0.1 Hz, 0.5 Hz, 1 Hz, 5 Hz, 10 Hz, and 25 Hz), and five values of testing temperatures (i.e., 10 °C, 20 °C, 30 °C, 40 °C, and 50 °C).

### 2.4. Instrumentation

The test samples were compacted with a Troxler’s Model 4140 Gyratory Compactor (TROXLER, Durham, NC, USA). The device could compact the sample with an angle of rotation from 0.5–2.0°. Usually, the machine will set the angle of 1.25°. The compression pressure was 600 kPa.

Cooper’s CRT NU 14 test equipment (Cooper, London, UK) was used to determine dynamic modulus (E*), according to AASHTO TP62. The device could power up to 20 kN with a gas supply of up to 7 Bar. The equipment can be loaded with frequencies varying from 0 to 30 Hz. The process of loading the axial strain of the test piece was determined by Linear Variable Differential Transformers (LVDTS) (Cooper, London, UK). All laboratory samples and equipment were placed in the Cooper temperature chamber with a tolerance of ±0.1 °C.

## 3. Machine Learning Approaches

### 3.1. Artificial Neural Network (ANN)

The ANN is one of the most popular and effective machine learning models used widely to deal with a lot of complex real-world problems, including problems related material sciences such as self-compacting concrete strength prediction [22], modeling in the compression of austenitic stainless steel [23], and concrete strength prediction [22]. The main principle of the ANN algorithm is based on the biological neural network of the brain of humans. The structure of the ANN consists of three components, such as input variables, output variables, and activation functions (hidden layers). In the modeling, the activation functions used in hidden layers are trained to analyze and discover the relationship between input variables and output variables for prediction and assessment of the problems. In this study, sigma functions—a popular activation function used in the ANN—were selected to predict the dynamic modulus of warm mix asphalt with high reclaimed asphalt pavement. Furthermore, the Levenberg–Marquardt (LM) algorithm was used to optimize the neural networks’ learning process. The final ANN model is herein denoted as ANN-LMN.

### 3.2. Support Vector Machine (SVM)

The SVM was first proposed by Vapnik [24], and is one of the most effective machine learning algorithms as it has the capacity to minimize the outliers and noise [25]. The main principle of the SVM is to transfer the original input space into a high-dimensional feature space using a hyperplane [26], which is determined during the training process. The SVM function is expressed by the following Equation (1):

(1) y=f(x)=wϕ(x)+c

where w is the weight matric, *x* = *x*_i_ is input variables, *y* is the output variable, *c* is the bias of the model, and ϕ(x) is defined as an activation function.

### 3.3. Ensemble Boosted Trees (Boosted)

Decision trees (DT) and boosting techniques were combined to form a hybrid model, namely the Boosted. Out of these, DT are utilized to analyze the relationship between output and input variables using recursive dual separations, whereas the boosting technique is used to associate many individual DT models to construct the hybrid model with improved performance [27]. In the hybrid DT, the merits of tree-based techniques can be highlighted as (i) a proper variable can be chosen to match appropriate functions, (ii) the random boosting is used to fit this model with various amounts of data, and (iii) model averaging is used to reduce both bias and variance of this model [28]. 

### 3.4. Gaussian Process Regression (GPR)

The GPR is a well-known probabilistic, nonparametric technique used to solve nonlinear regression problems [29]. Initially, GPR was proposed to solve the limitation of the relevance vector machine (RVM). It can be utilized to define prior distributions over latent functions based on a Bayesian learning algorithm. It is also based on the assumption that Gaussian is the joint probability distribution of model outputs. Many advantages of GPR compared with other machine learning models are hyperparameter estimation and uncertainty analysis or estimation [30]. Thereby, the performance of the predictive model using GPR is less affected by subjectivity [31].

### 3.5. Quality Assessment Criteria

In this study, various popular quantitative statistical indexes, namely mean absolute error (MAE), root mean square error (RMSE), and correlation coefficient (R) were used to validate and compare the performance of different machine learning models. A detailed description of these indices is presented in previously published works [32,33,34,35,36,37,38,39]. Their calculation can be carried out by using the following Equations (2)–(4) [40,41,42,43]:

(2) $$\mathrm{MAE}=\frac{1}{k}\sum_{i=1}^{k}\left(p_{i}-{\;p\;¯}_{i}\right)$$

(3) $$\mathrm{RMSE}\;=\;\sqrt{\frac{1}{k}\sum_{i=1}^{k}{\left(p_{i}-{\bar{p}}_{i}{}_{i}\right)}^{2}}$$

(4) $$R=\sqrt{1-\frac{\sum_{i=1}^{k}{\left(p_{i}-{\bar{p}}_{i}\right)}^{2}}{\sum_{i=1}^{k}{\left(p_{i}-\bar{p}\right)}^{2}}}$$

where $p_{i}$: actual output, ${\bar{p}}_{i}$: predicted output, $\bar{p}$: mean of the $p_{i}$ and *k:* number of samples.

## 4. Results and Discussion

This section is dedicated to the presentation of the results obtained in this study. It is worth noticing that the present dataset contains 300 instances. The result of each instance was derived by taking the average value of six experimental results, making a total number of 1800 tests performed. The dataset was next divided into two parts, the training dataset served for the development of the machine learning algorithms (containing 70% of data, or 210 samples), and the testing dataset used to assess the performance of the constructed algorithms (including 30% (the remaining data), or 90 samples). The results presented herein reflect the best performance of the machine learning algorithms with the highest values of R, and the lowest values of RMSE and MAE.

This section is presented in the following steps. The experimental results are first provided, followed by the application of machine learning methods to predict the values of dynamic modulus (E*). In this step, the performance of four algorithms, namely ANN-LMN, SVM, GPR, and Boosted, are evaluated and compared in function of different statistical criteria. Finally, the best predictor was used to interpret the results, and to perform the parametric study to reveal the relationship and dependence of the predicted output on the input variables.

### 4.1. Experimental Results

**Figure 4:**
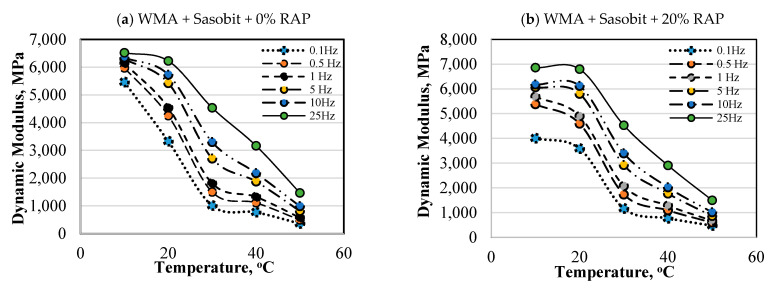

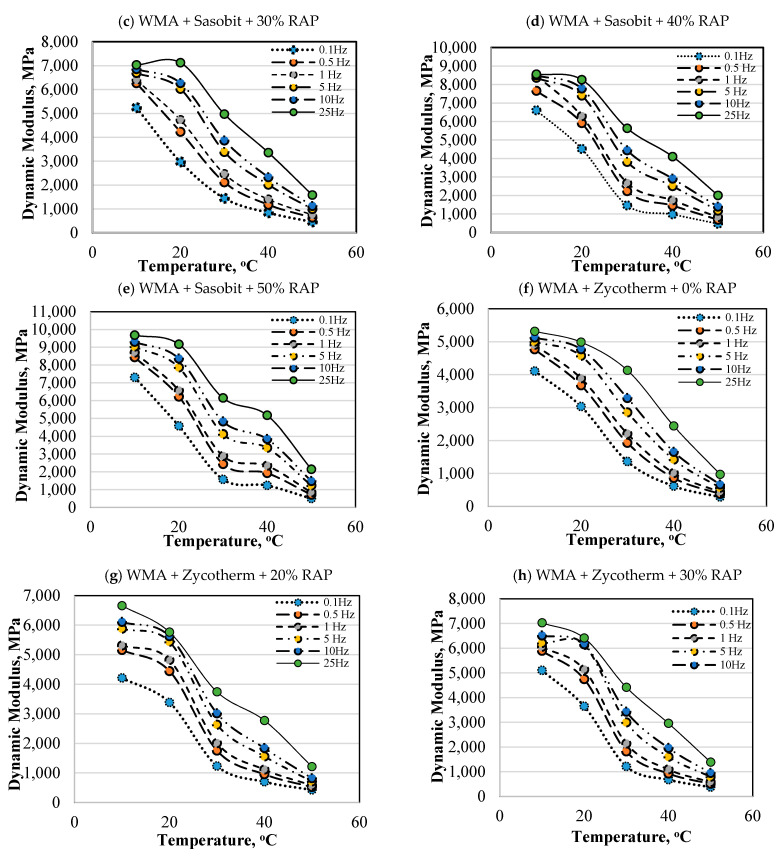

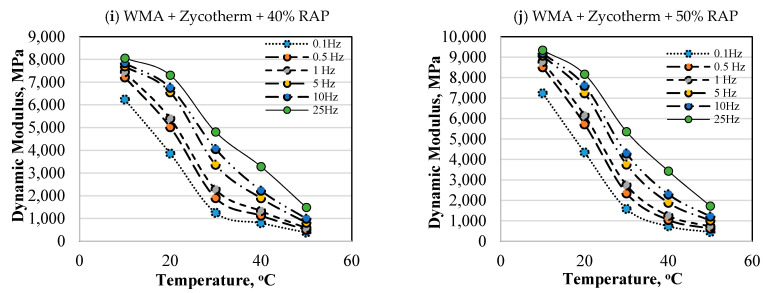
Experimental results of Dynamic Modulus (E*) vs. temperature in function of different asphalt mixtures and RAP contents.

The test results of the sample groups at different test temperatures and frequencies, following the ASTM E187, are shown in Figure 4. It could be observed that at the same test temperature, the value of dynamic modulus (E*) decreased as the frequency decreased from 25 Hz to 0.1 Hz. Another observation could also be noticed for the test temperatures of 40 °C and 50 °C, as there was an important difference in dynamic modulus (E*) between test frequency 25 Hz compared to other frequencies (i.e., from 10 Hz to 0.1 Hz). However, with the frequencies of 10 Hz or 5 Hz, 0.1 Hz, there was no significant difference between the E* values. This result clearly reflected the viscosity elasticity characteristic of asphalt mixture at high temperatures. Regarding the effect of RAP content, it was shown that when the RAP content increased from 0% to 20%, 30%, 40%, and 50%, the value of E* increased by 5.4%, 8.8%, 18.5%, and 11.8%, respectively. Furthermore, due to the higher hardening properties of Sasobit compared to Zycotherm, the mixtures with Sasobit had an 11.4% higher value of E* considering the same RAP content.

In general, the test results showed that when the test temperature increased, the E* tended to decrease at all test frequencies. Moreover, at the same test temperature, the E* test results tended to increase when the test frequency increased from 0.1 Hz to 25 Hz.

### 4.2. Prediction Performance of Machine Learning Models

**Figure 5:**
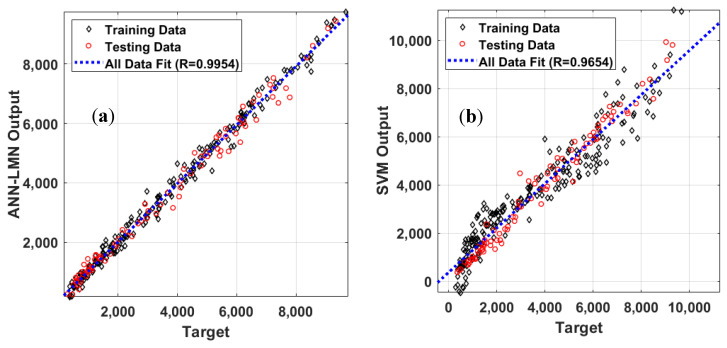

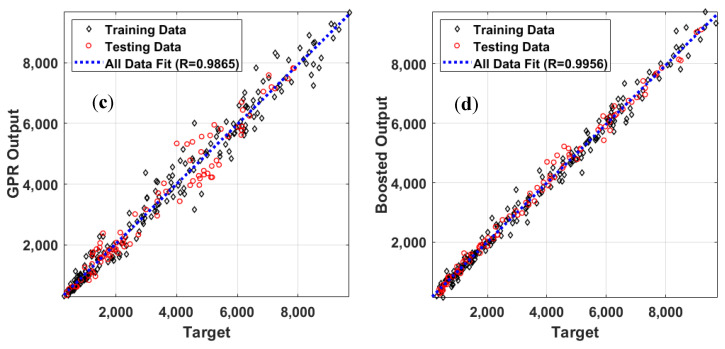
Graphs of correlation between measured E* versus predicted E* using (**a**) ANN-LMN, (**b**) Support vector mechanism (SVM), (**c**) Gaussian process regression (GPR), and (**d**) Boosted.

The performance and effectiveness of four machine learning models are evaluated in this section. The prediction performance in a regression form is shown (Figure 5) for the training, testing, and all datasets. A summary of the corresponding information is indicated in Table 5. It is worth mentioning that the results presented herein were transformed into the normal range.

With respect to the training parts, the ANN-LMN model demonstrated better performance, yielding a correlation of R = 0.9959, RMSE = 225.99, and MAE = 171.81. The Boosted model produced an intermediate accuracy (R = 0.9953, RMSE = 248.54 and MAE = 179.82), followed by the GPR model (R = 0.9880, RMSE = 408.74 and MAE = 292.05), and the SVM algorithm (R = 0.9565, RMSE = 758.50 and MAE = 623.10). 

**Figure 6:**
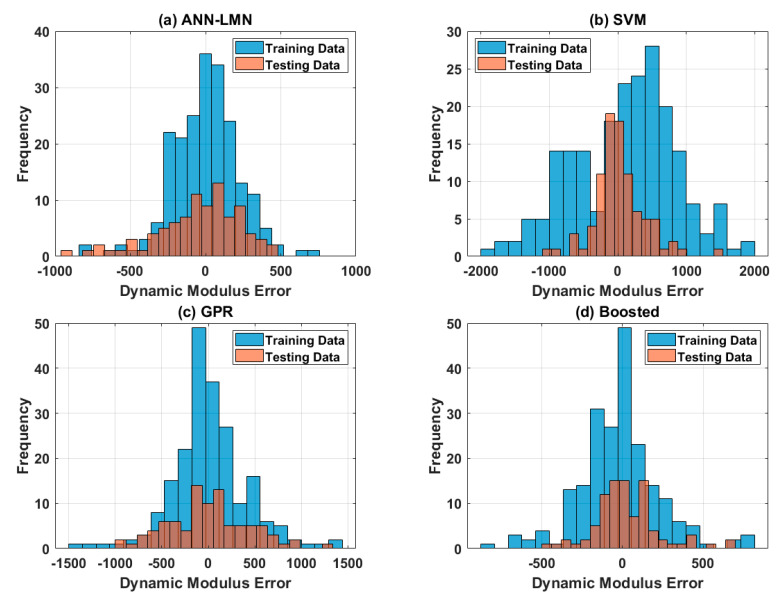
Graphs of error frequency between the predicted and experimental values for (**a**) ANN-LMN, (**b**) SVM, (**c**) GPR, and (**d**) Boosted.

Considering the testing datasets, the Boosted model yielded the best prediction results with respect to all statistical measurements (i.e., R = 0.9967, RMSE = 203.84, MAE = 144.68), followed by ANN-LMN, SVM, and GPR (Table 5). The R value of the SVM was slightly higher than the ANN-LMN and the Boosted, whereas those of RMSE and MAE were higher compared with the ANN-LMN and the Boosted (Table 5). The regression graphs of the four proposed models are plotted in Figure 6. As can be seen, the predicted outputs were in good agreement with the experimental values

### 4.3. Mapping of the Relationship of Dynamic Modulus (E*) with Input Variables 

**Figure 7:**
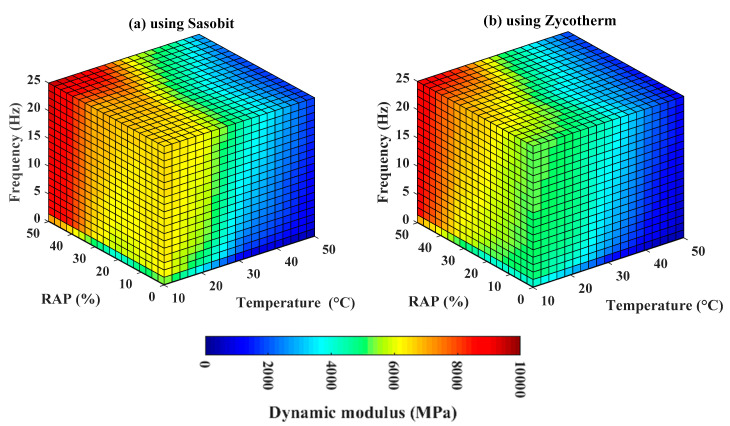
Box map sensitivity analysis at different %RAP contents, frequencies, and temperatures using (**a**) Sasobit; and (**b**) Zycotherm.

**Figure 8:**
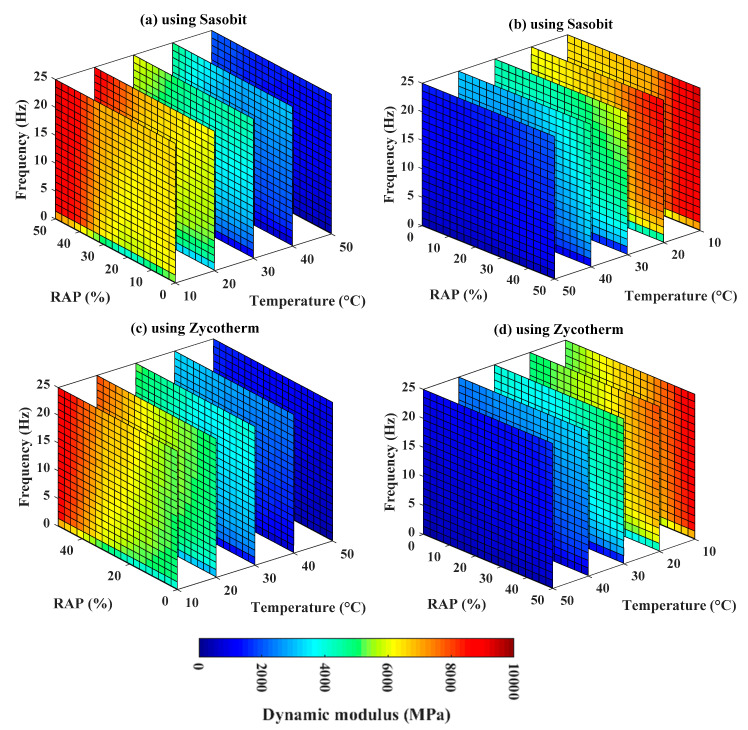
Box map sensitivity analysis at different %RAP contents, frequencies and temperatures varying from 10 °C to 50 °C using sliced mode with (**a**) Sasobit, standard view; (**b**) Sasobit, opposite view; (**c**) Zycotherm, standard view; and (**d**) Zycotherm, opposite view.

As identified in the previous section, the Boosted model exhibited the best prediction performance. Therefore, it could be used as a continuous function for mapping dynamic modulus and input variables such as temperature, RAP, frequency, and additives used (i.e., Sasobit and Zycotherm), within the ranges of the input variables adopted in this study. Figure 7a,b show the corresponding box maps using Sasobit and Zycotherm technology, respectively. For illustration purposes, Figure 8 presents the corresponding box maps sliced at different temperatures, such as 10 °C, 20 °C, 30 °C, 40 °C, and 50 °C, and using Sasobit and Zycotherm additives, respectively (at two opposite viewpoints). In these figures, the dynamic modulus (E*) was highlighted in the same color scale between 0 and 10,000 MPa. The results showed that the dynamic modulus (E*) exhibited a nonlinear relationship with all input variables, regardless of the technology used. Moreover, as deduced in Figure 8, a similar trend of variation of dynamic modulus (E*) was observed using Sasobit and Zycotherm technology.

For comparison purposes, an indicator called Δ, was introduced and expressed as below Equation (5):

(5) $$\Delta =\frac{E_{\mathrm{Zycotherm}}^{*}-E_{\mathrm{Sasobit}}^{*}}{E_{\mathrm{Sasobit}}^{*}}\times 100$$

**Figure 9:**
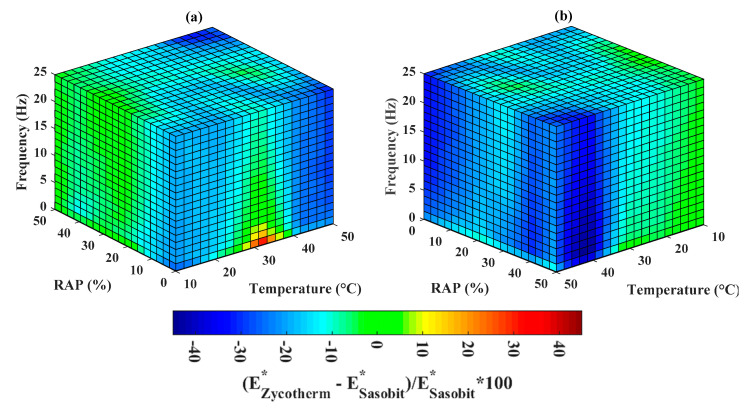
Box map comparison analysis for the two technologies in varying %RAP, frequencies and temperatures, (**a**) standard view; and (**b**) opposite view.

The corresponding box map of Δ is presented in Figure 9, with two opposite viewpoints. The color scale is in the range of (−45, 45) (%). Clearly, it can be seen that the dynamic modulus (E*) using Zycotherm technology could be equal (green zone), higher (red zone), or smaller (blue zone) than that using Sasobit technology. However, such variation is local. The magnitude of the indicator Δ could be increased by up to 45%. For instance, at 30 °C, the lowest frequency and lowest value of RAP, Zycotherm technology provided a dynamic modulus (E*) 45% larger than that acquired with Sasobit technology.

**Figure 10:**
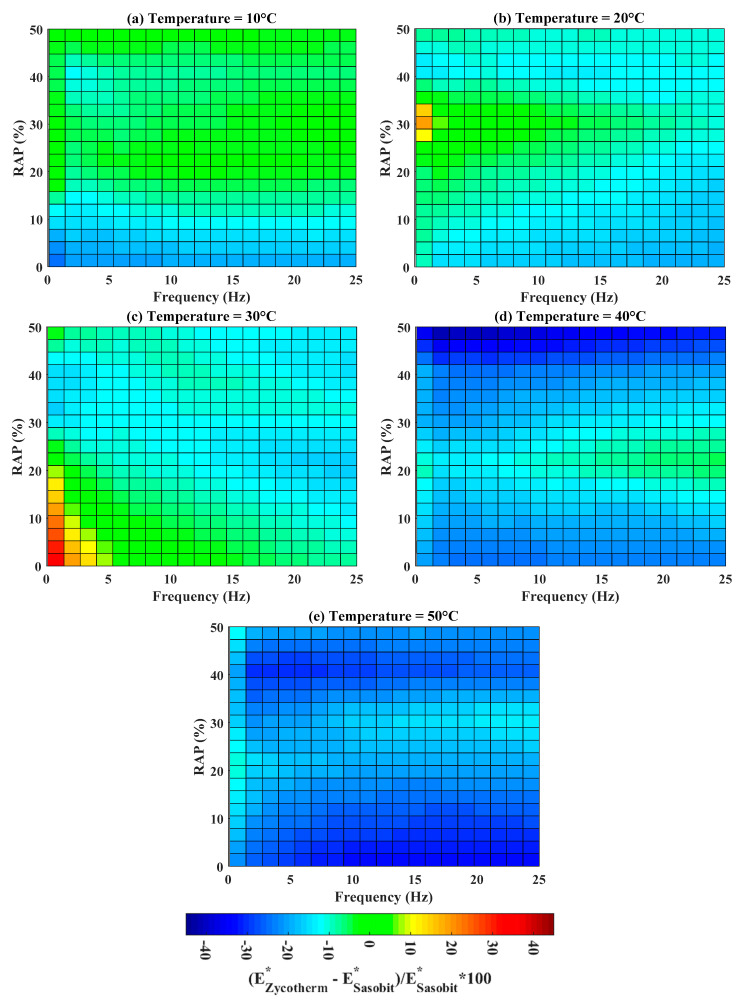
Box map sensitivity analysis and comparison of the two technologies at different %RAP, frequencies and temperatures (**a**) T = 10 °C; (**b**) T = 20 °C; (**c**) T = 30 °C; (**d**) T = 40 °C; and (**e**) T = 50 °C.

The indicator Δ was next investigated at five temperatures, such as 10, 20, 30, 40, and 50 °C, as shown in Figure 10. Regarding Δ at temperature of 10 °C, it corresponded to the zone of highest dynamic modulus (up to 10,000 MPa, illustrated in Figure 8); both Zycotherm and Sasobit technologies provided similar values of dynamic modulus for RAP higher than 10%, as Δ is in the range of (−10, 10) (%). For an RAP content lower than 10%, Sasobit technology was better.

It is observed in Figure 10a–e that the blue surface of Δ was increased while increasing the temperature (i.e., the similitude zone between Zycotherm and Sasobit was reduced, except for a small red zone in Figure 10c at low frequency and RAP). Such a result led to a better performance of Sasobit technology than Zycotherm. Notably, at 50 °C, the dynamic modulus using Sasobit technology was higher than that using Zycotherm at all frequencies and % RAP.

**Figure 11:**
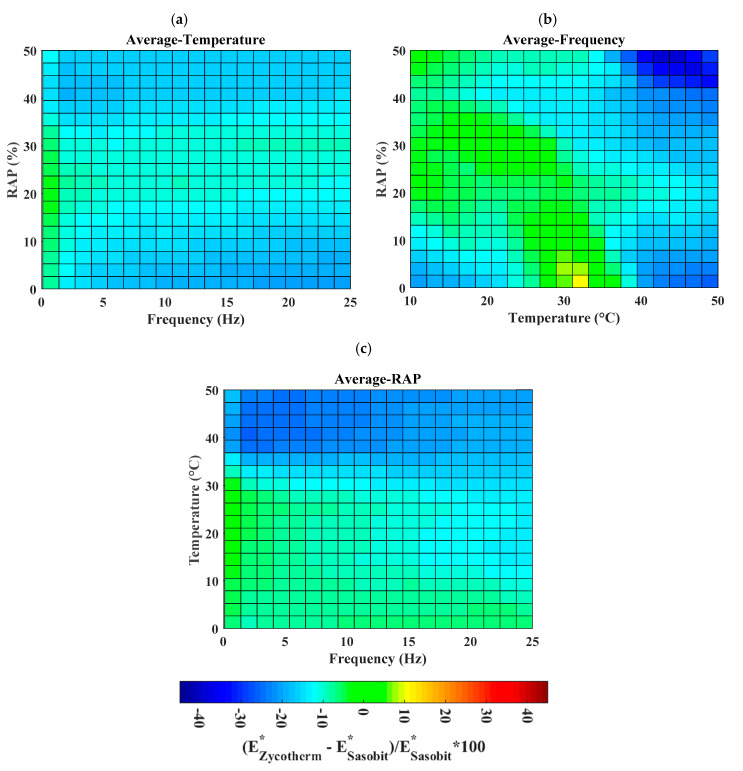
Surfaces of average values of Δ in function of (**a**) temperature, (**b**) frequency, and (**c**) RAP, respectively.

In order to obtain a general view on the difference between Sasobit and Zycotherm, the average values of Δ were calculated in function of the temperature, frequency, and %RAP. Figure 11a–c present the surface of average Δ in function of the average values of these three features. Taking the average value of temperature, the difference seemed significant, with the value of frequency superior to 2 Hz and at low and high % RAP (Figure 11a). On the contrary, taking the average frequency as the chosen feature, the two additives exhibited a significant effect at high temperature (i.e., higher than 40 °C) (Figure 11b). Finally, with the average % RAP, higher values of the temperature (i.e., higher than 40 °C) led to an important distinction between Zycotherm and Sasobit (Figure 11c).

Overall, E* depends on many factors, which can be categorized into two groups: (i) testing condition (i.e., testing temperature, testing frequency), and (ii) the mixture content (i.e., coarse and fine aggregates, type of aggregates, binder content, binder type, the RAP content). It is worth noticing that in this study, the air void, aggregates gradation, bitumen type and content were kept constant. Two main reasons were considered. First, most of the asphalt mixtures used in Vietnam and many other countries are designed with a targeted and typical value of V_a_ of 7 ± 0.5%. Second, regarding the mix design procedure, 300 mixtures in the database were already optimized. It means that in order to construct a database of 300 instances, many other non-optimum samples had been fabricated. However, due to the E* testing cost and testing time required, only 300 optimized mixtures were finally performed. Therefore, a particular interest of the present study is to extend the input variables by performing an investigation on the effect of other parameters on the predicted values of E*.

In short, this preliminary investigation allowed the attainment of quantitative maps of dynamic modulus in function of input variables. Moreover, as a machine-learning technique, the Boosted model can be extended to learn in a broader range of input variables in further researches.

## 5. Conclusions and Outlook

The present work proposed a cost-effective alternative approach, based on machine learning algorithms (ANN-LMN, SVM, GPR, and Boosted), to predict the dynamic modulus (E*) of WMA with high RAP content. For this purpose, a database consisting of 300 experimental tests was constructed, analyzed, and used for the development of four machine learning models. Concerning the experimental study, the following observations can be drawn: (i) the use of both additives resulted in a significant reduction in mixing and constructing temperatures so that WMA technology could be favorably applied compared with traditional HMA technology, (ii) the value of dynamic modulus (E*) of WMA mixtures using Sasobit and Zycotherm additives had a close relationship with the testing temperatures and frequencies (i.e., the dynamic modulus (E*) values decreased with an increase of the testing temperature and decrease of the testing frequency), (iii) the value of dynamic modulus (E*) improved with the incorporation of RAP, and the effect was more pronounced when RAP content was increased to 50%, (iv) the use of Sasobit additive resulted in a better value of dynamic modulus (E*) in comparison with Zycotherm. Concerning the results of the simulation of dynamic modulus (E*), the Boosted algorithm was found as the best predictor compared with those obtained by ANN-LMN, SVM, and GPR (i.e., R = 0.9954, R = 0.9654, R= 0.9865, and R = 0.9956 for all dataset using ANN-LMN, SVM, GPR, and Boosted, respectively). Finally, the Boosted “machine learning black-box” was used to derive a map that related the dynamic modulus (E*) in function of input variables, namely the testing temperature, frequency, RAP content, and additives. The proposed maps could assist researchers, engineers with a more in-depth understanding of the mixing process, and the working mechanism of WMA containing high RAP content. Furthermore, it is recommended that the proposed models should be applied and validated in other new datasets considering different types of RAP, aggregates, and bitumen in the mixture to extend the range of possible applications.

## Figures and Tables

**Table 1:** Properties of aggregates and mineral filler.

Properties	Agg._12.5_	Agg._9.5_	Agg._9.5_	Filler	Specification
Bulk Specific Gravity (G_sb_)	2.735	2.725	2.702	2.712	AASHTO T84, 85
App.Specific Gravity (G_sa_)	2.751	2.752	2.740	-	AASHTO T84, 85
Absorption, %	0.207	0.365	0.517	-	AASHTO T84, 85
Clay and dust content, %	0.35	0.67	-	-	ASTM C142
Flat and elongation, %	11.26	8.41	-	-	ASTM D4791
Fineness modulus	-	-	3.48	-	ASTM C33
Los Angeles, %	23.48	-	-	-	ASTM C131
Sand equivalent value	-	-	73.9	-	AASHTO T176
Plasticity index	-	-	-	1.09	ASTM D4318

**Table 2:** Properties of bitumen 60/70, bitumen 60/70 with Sasobit, and bitumen 60/70 with Zycotherm.

Condition	Properties	Bitumen 60/70 (Virgin)	Bitumen 60/70 with Sasobit	Bitumen 60/70 with Zycotherm	Specification
Unaged	Penetration at 25 °C, 0.1 mm	66	52	64	ASTM D 5
	Flash point, °C	253	247	259	ASTM D 92
	Softening point, °C	49.5	69.8	50.7	ASTM D 36
	Ductility at 25 °C, cm	>100	>100	>100	ASTM D 113
	Rotational Viscometer, Pa.s	0.385	0.237	0.316	AASHTO T316
	G*/sinδ at 64 °C, kPa	1.15	2.96	1.06	AASHTO T315
Short-term aged (RTFO)	G*/sinδ at 70 °C, kPa	2.62	6.497	3.439	

**Table 3:** Properties of recovered reclaimed asphalt binder.

Properties	Value	Specification
Penetration at 25 °C, 0.1 mm	25.4	ASTM D 5
The softening point, °C	76.7	ASTM D 36
Viscosity at 135 °C, Pa.s	2.134	AASHTO T316
G*/sinδ at 82 °C, kPa	1.1	AASHTO T315

**Table 4:** Composition and properties of different mixtures.

	WMA with RAP + Zycotherm					WMA with RAP + Sasobit				
Mix Composition/Mix Content										
RAP	0	19.0	28.5	37.9	47.4	0.0	19.0	28.5	37.9	47.4
Agg._12.5_	19.2	9.5	9.5	6.6	6.6	19.2	9.5	9.5	6.6	6.6
Agg._9.5_	28.8	20.0	16.1	14.2	8.5	28.8	20.0	16.1	14.2	8.5
Agg._4.75_	43.2	42.8	38.0	33.2	30.3	43.2	42.8	38.0	33.2	30.3
Filler	3.8	3.8	2.8	2.8	1.9	3.8	3.8	2.8	2.8	1.9
P_b(RAP)_	0.0	0.8	1.1	1.5	1.9	0.0	0.8	1.1	1.5	1.9
P_bn_	5.0	4.2	4.0	3.7	3.4	5.0	4.2	4.0	3.7	3.4
Total	100	100	100	100	100	100	100	100	100	100
**Properties of Mixtures**										
V_a_ (%)	4.5	3.9	4.1	4.1	4.3	3.9	4.0	4.1	3.8	4.1
VMA (%)	15.7	14.1	14.1	14.3	14.3	14.3	14.5	14.3	14.1	14.1
VFA (%)	71.5	72.1	70.7	70.9	70.1	72.4	71.0	71.0	71.4	70.6
P_ba_ (%)	0.3	0.72	0.70	0.95	1.05	0.44	0.72	1.04	1.22	1.48
P_be_ (%)	4.7	4.32	4.44	4.30	4.31	4.58	4.32	4.11	4.04	3.90
P_0.075_/P_be_	0.8	0.85	0.83	0.86	0.86	0.81	0.00	0.90	0.91	0.95
MS (kN)	11.3	13.83	15.03	16.06	16.64	13.45	14.97	16.37	18.11	19.10
MF (mm)	3.5	3.62	3.58	3.58	3.56	3.27	3.62	3.58	3.56	3.61

where V_a_ = Air voids; VMA = Voids in the mineral aggregate; VFA = Voids filled with asphalt; P_ba_ = Absorbed binder content; P_be_ = Effective binder content; P_0.075_/P_be_ = Dust-to-binder ratio; MS = Marshall Stability; MF = Marshall Flow; P_b(RAP)_ = content of asphalt binder of RAP (%); P_bn_ = virgin asphalt binder content.

**Table 5:** Summary information of different machine learning algorithms.

Dataset	Criterion	ANN-LMN	SVM	GPR	Boosted
Training	RMSE	225.99	758.50	408.74	248.54
	MAE	171.81	623.10	292.05	179.82
	R	0.9959	0.9565	0.9880	0.9953
Testing	RMSE	283.38	376.29	432.82	203.84
	MAE	215.15	265.50	333.68	144.68
	R	0.9944	0.9989	0.9819	0.9967
All	RMSE	244.63	667.23	416.11	236.02
	MAE	184.82	515.82	304.54	169.28
	R	0.9954	0.9654	0.9865	0.9956
